# Supplementary material for: Predictors of educational failure at 16 and 19 years of age—SESBiC longitudinal study
Source: PLoS One. 2023 Jan 11;18(1):e0279531. doi: 10.1371/journal.pone.0279531 (PMC9833512; doi:10.1371/journal.pone.0279531)
Supplement: Attachment — Submitted filename: PlosOne response letter.docx [file pone.0279531.s001.docx]

Dear Editor,

First, I would like to thank you for the extension due to my illness (which turned out to be Covid-19). I do acknowledge that this may be a bit troublesome, but nevertheless much appreciated by me!

Below I have tried to clarify the remaining questions.

Best regards,

Marie Bladh

Dear Dr. Bladh,

We've checked your submission and before we can proceed, we need you to address the following issues:

1. We note that the grant information you provided in the ‘Funding Information’ and ‘Financial Disclosure’ sections do not match.

*I must admit I am a bit confused here. In the submission guidelines it states:* Do not include funding sources in the Acknowledgments or anywhere else in the manuscript file. Funding information should only be entered in the financial disclosure section of the submission system.

*Thus, I wonder how there can be discreapancies.*

When you resubmit, please ensure that you provide the correct grant numbers for the awards you received for your study in the ‘Funding Information’ section.

2. We note that you have included the phrase “data not shown” in your manuscript (Page 10). Unfortunately, this does not meet our data sharing requirements. PLOS does not permit references to inaccessible data. We require that authors provide all relevant data within the paper, Supporting Information files, or in an acceptable, public repository. Please add a citation to support this phrase or upload the data that corresponds with these findings to a stable repository (such as Figshare or Dryad) and provide and URLs, DOIs, or accession numbers that may be used to access these data. Or, if the data are not a core part of the research being presented in your study, we ask that you remove the phrase that refers to these data.

*As the section where the “data not shown” statement is not essential to the manuscript, I have removed this sentence (pg 10).*

3. We note your current data availability statement:

"Data cannot be shared publicly because this was not included in the regional ethics committees permission at the start of the study in 1995."

Please note that PLOS requires data to be made available on request if there are ethical or legal restrictions on sharing the data. Before we proceed, we’ll require some additional information to ensure your submission adheres to the PLOS policy on acceptable data access restrictions: [https://eur01.safelinks.protection.outlook.com/?url=https%3A%2F%2Fjournals.plos.org%2Fplosone%2Fs%2Fdata-availability%23loc-acceptable-data-access-restrictions&amp;data=05%7C01%7Cmarie.bladh%40liu.se%7Ca632397252a741adbaa308da5a018fc1%7C913f18ec7f264c5fa816784fe9a58edd%7C0%7C0%7C637921259999153592%7CUnknown%7CTWFpbGZsb3d8eyJWIjoiMC4wLjAwMDAiLCJQIjoiV2luMzIiLCJBTiI6Ik1haWwiLCJXVCI6Mn0%3D%7C3000%7C%7C%7C&amp;sdata=%2BZtA92777nixB1okMY8hxj0abD3axLO%2FLHxXtqChz80%3D&amp;reserved=0](https://eur01.safelinks.protection.outlook.com/?url=https%3A%2F%2Fjournals.plos.org%2Fplosone%2Fs%2Fdata-availability%23loc-acceptable-data-access-restrictions&data=05%7C01%7Cmarie.bladh%40liu.se%7C53c97117422f4000f0a008da5fb12bf0%7C913f18ec7f264c5fa816784fe9a58edd%7C0%7C0%7C637927511803437603%7CUnknown%7CTWFpbGZsb3d8eyJWIjoiMC4wLjAwMDAiLCJQIjoiV2luMzIiLCJBTiI6Ik1haWwiLCJXVCI6Mn0%3D%7C3000%7C%7C%7C&sdata=%2BCvV41CIHIs9pLeJpiTgub5KH38t4G07tP%2BlagBd4m0%3D&reserved=0).

a) Please confirm whether there are legal or ethical restrictions on sharing your data publicly.

b) If legal or ethical restrictions apply, please provide all necessary instructions and non-author contact information (preferably email) for a data access committee, ethics committee, or other institutional body that other researchers would require to request access to your data. **Note that it is not acceptable for an author to be the sole named individual responsible for ensuring data access.**

c) If there are no legal or ethical restrictions on sharing your data publicly, please upload the minimal anonymized data set necessary to replicate your study findings as either Supporting Information files, or to a stable public repository. If you upload your data to a repository, please also provide the relevant URLs, DOIs, or accession numbers for other researchers to access your data directly. For a list of recommended public repositories, please see [https://eur01.safelinks.protection.outlook.com/?url=https%3A%2F%2Fjournals.plos.org%2Fplosone%2Fs%2Frecommended-repositories&amp;data=05%7C01%7Cmarie.bladh%40liu.se%7Ca632397252a741adbaa308da5a018fc1%7C913f18ec7f264c5fa816784fe9a58edd%7C0%7C0%7C637921259999153592%7CUnknown%7CTWFpbGZsb3d8eyJWIjoiMC4wLjAwMDAiLCJQIjoiV2luMzIiLCJBTiI6Ik1haWwiLCJXVCI6Mn0%3D%7C3000%7C%7C%7C&amp;sdata=OdzXHAx%2B7ljnuhvsUPVY%2BKVjV5lmZeO1VWgCNfND%2BJg%3D&amp;reserved=0](https://eur01.safelinks.protection.outlook.com/?url=https%3A%2F%2Fjournals.plos.org%2Fplosone%2Fs%2Frecommended-repositories&data=05%7C01%7Cmarie.bladh%40liu.se%7C53c97117422f4000f0a008da5fb12bf0%7C913f18ec7f264c5fa816784fe9a58edd%7C0%7C0%7C637927511803437603%7CUnknown%7CTWFpbGZsb3d8eyJWIjoiMC4wLjAwMDAiLCJQIjoiV2luMzIiLCJBTiI6Ik1haWwiLCJXVCI6Mn0%3D%7C3000%7C%7C%7C&sdata=bKiUui7KyVhASRNGQS%2FAizKzvrkBmg45qDXfUPmAkk0%3D&reserved=0).

Once we receive this information, we will update your data availability statement on your behalf.

*Thank you for this kind offer to help me updating the availability statement. This is how we have formulated the availability statement previously: “*The data in the current study can, due to the legal agreement accepted upon delivery of data from the National Board of Health and Welfare, only be shared on a group level upon request by a researcher. For requests contact M. Bladh, MD or G. Sydsjö, Professor, gunilla.sydsjo@regionostergotland.se.”

*Would this phrasing be acceptable?*
