# Supplementary material for: Predictors of educational failure at 16 and 19 years of age—SESBiC longitudinal study
Source: PLoS One. 2023 Jan 11;18(1):e0279531. doi: 10.1371/journal.pone.0279531 (PMC9833512; doi:10.1371/journal.pone.0279531)
Supplement: Attachment — Submitted filename: Response to reviewers 221103.docx [file pone.0279531.s002.docx]

Dear Editor,

Thank your letting us further improve our manuscript. We have tried our utmost to answer and resolve each of the problems addressed.

Looking forward to your decision.

Best regards,

Marie Bladh

PONE-D-21-22136R1

Predictors of educational failure at 16 and 19 years of age – SESBiC longitudinal study.

PLOS ONE

Dear Dr. Bladh,

Reviewer has suggested a few points to improve the paper and I would appreciate if you could address and include those concerns in the revised mansucript by Nov 4, 2022. 

Please include the following items when submitting your revised manuscript:

- A rebuttal letter that responds to each point raised by the academic editor and reviewer(s). You should upload this letter as a separate file labeled 'Response to Reviewers'.
- A marked-up copy of your manuscript that highlights changes made to the original version. You should upload this as a separate file labeled 'Revised Manuscript with Track Changes'.
- An unmarked version of your revised paper without tracked changes. You should upload this as a separate file labeled 'Manuscript'.

Reviewer #1: Predictors of educational failure at 16 and 19 years of age – SESBiC longitudinal study.

In this study the authors present analyses from a longitudinal study on children born in the mid-1990s in southern Sweden and factors associated with school grades. The study is interesting and apart from some minor typing errors, the it is well written and easy to comprehend. I have, however, some comments.

Major comments
1. There is a huge difference between “significant” and “statistically significant”. In the conclusion of the abstract, the authors claim that ***“Identifying mothers smoking during pregnancy or with high level of stressors as well as children with externalizing behaviour problems to provide guidance and support is very important as these two factors appear to be associated with future study performance in both lower and upper secondary school”.*** Although the results are interesting, they do not support such a claim. For example, only 69 of 339 children exposed to maternal smoking in utero were ineligible for upper secondary school. The positive predictive value was thus only 0.21. Since there are no tests in the study of the precision with which educational failure can be predicted on the basis of the covariates under study, I think the authors should abstain from drawing clinical conclusions that are not supported by their results.

*ANSWER: The wording of the conclusion has been rephrased. Hopefully this change is satisfactory, and better reflect the findings in the current study.*

2. In the Discussion, it is suggested that the association between maternal smoking during pregnancy and offspring’s school performance is a casual one. This may definitely be so, but given that maternal IQ and smoking during pregnancy also have been shown to correlate, the association found by the authors could have other explanations. Since correlation does not prove causation, the authors should be more prudent in the interpretation of their data.

*ANSWER: We agree with this comment and the interpretation, and the discussion of the results has been rephrased to better reflect the actual results. Additional text has been added to add additional possible explanations.*

3. In Table 4, missing values appears to have huge effects on the analysis since 7 out of 18 analysis cannot be performed because of missing data.

*ANSWER: It is not missing data that are the cause of NA. The variables presented are the results from stepwise regression models and NA in this case implies not being included in the modelling (as only statistically significant variables from table 1-3 were considered, separately, for each outcome). In the footnotes to the table, variables included in each of the models are described. However, given the misunderstanding, we have tried to further clarify the models.*

How many study subjects are included in the final models? Given the appearance of the table, I would assume this number to be rather low. Have the authors tried to address this problem?

*ANSWER: The final models described in table 4, included a total of 670 individuals. This lowered number is due to participants not having participated in each of the follow-ups (i.e., a participant may have chosen to participate in the 3-year follow-up and to not participate in the 12-year follow-up, and then return in the next follow-up). This reduces the validity of using e.g., imputation of missing data. The lowered number of participants is also a reason to why we chose to, initially, model each table separately in order to identify variables of importance in a larger population.*

Minor comments:
1. In the abstract, the abbreviation “CBCL” is not explained. For those not in the field, this makes the abstract less comprehensible.

*Answer: We agree with the reviewer and thus the abbreviation has been replaced with Child Behavior Checklist.*
2. Is it really necessary to introduce the abbreviation GPA?

*Answer: Since it is frequently used, we actually think that using GPA instead of grade point average simplifies the reading of the paper.*
3. In the methods, second paragraph, the sentence “….yet viewed as a an education one must complete” suggests, according to my interpretation, that upper secondary schooling is more a question of social and peer pressure than about, for example, increasing the individuals chances of finding a job in the future. Even though I agree with the authors to a large extent, I find this phrasing a little unidimensional.

*Answer: Thank you for this comment. We see your point and have therefore removed “yet viewed as a an education one must complete” from the sentence as the message regarding societal expectancy is mentioned in the next sentence.*
4. In the Study Population section, “five municipalities in southern Sweden” is a little vague. Is there a thought behind this way of presenting the study population?

*Answer: No, there is no thought behind this presentation more than not finding it useful to provide the names of the municipalities. The reason behind choosing these municipalities were that they were adjacent to one another and represented both urban and rural areas, providing a representative sample. This additional information has now been added to the manuscript.*
5. In the 3-year follow-up, the number of life events was dichotomized in <=8 and >8. Why this cut off?

*Answer: The cutoff of 8 was chosen because it represented the 90^th^ percentile. This information has been added to the manuscript.*
6. At the end of the same paragraph, there are minor typos (“percentilen” should be translated into English, and “child” is misspelt)

*Answer: Thank you for noticing the typos. These have been changed. The manuscript has now been scrutinized for additional typos.*
7. In the 12-year follow-up, there are also cut offs for dichotomization that are not explained.

*Answer: Thank you for this comment. The reasoning behind dichotomizing Rosenberg’s scale has now been added to the manuscript.*
8. Table 4. Does the table show all variables included in the model? This is not entirely clear.

Answer: As described in in the first paragraph of the section “Multiple logistic regression models”, only variables that were found to be statistically significant in tables 1-3 were considered in the models. This information can now also be found in the statistics. NA has now been replaced with “Not included” to highlight that these variables were not considered in the model.
